# Occupational Practices and Hazards of Rural Livestock Keepers in Uganda: A Cross-Sectional Study

**DOI:** 10.24248/EAHRJ-D-17-00139

**Published:** 2018-04-01

**Authors:** Julianne Meisner, Kellie Curtis, Thomas Graham, Peter Rabinowitz

**Affiliations:** a Department of Epidemiology, University of Washington, Seattle, Washington, USA; b Center for One Health Research, University of Washington, Seattle, Washington, USA; c Veterinarians Without Borders, Davis, California, USA; d Department of Environmental and Occupational Health Sciences, University of Washington, Seattle, Washington, USA

## Abstract

**Objective:**

In Uganda, 70% of rural poor rely on livestock for subsistence, to meet social obligations, and to insure against disaster. Livestock farming in Africa is in a state of transition from traditional management systems toward intensified modern systems, calling into question the future of traditional systems. To inform this debate, we conducted a survey in Moyo District, Uganda, to describe occupational practices and hazards of agropastoralist livestock keepers.

**Methods:**

Household surveys were administered to heads of household (N=49) from July to September 2016. Cross-sectional data were used to generate descriptive statistics for livestock-associated practices and exposures. Logistic regression was used to estimate odds ratios and Wald-type 95% confidence intervals for risk factors for injury, defined as any animal-related injury in the household in the past year. Risk factors studied were total number of male animals; number of male cattle, sheep/goats, and pigs; proportion male by herd size; herd size; and castration practices.

**Results:**

Adult men perform most livestock-associated tasks, while women, girls, and boys prepare meat, milk cattle, care for poultry, and dispose of waste. While 31 (63%) of households use professional veterinary services and most (n=28, 57.2%) are familiar with zoonoses, 25 (53.2%) do not believe sick animals may look healthy. Over 85.0% (n=41) of respondents routinely wash their hands, while only 31 (64.6%) use soap. Twenty-eight (57.0%) reported using personal protective equipment, while none used gloves or face protection. Most respondents had contact with animal waste “often”, and had contact with urine and blood “sometimes”. Six (12%) reported a needlestick injury while treating an animal, and 22 (45%) reported at least 1 injury from an animal. No significant association was found between the risk factors studied and animal injury, after adjustment for confounders.

**Conclusions:**

Occupational risks for female and young agropastoralists are distinct from those of men. Contact with potentially infectious material is common and current practices – handwashing without soap and low glove use – do little to prevent zoonotic transmission. While agropastoralists are familiar with zoonoses, subclinical infections may be missed. While no significant risk factors were identified for animal injury, both animal and needlestick injuries are common. As livestock agriculture intensifies, these hazards will become more pronounced; drivers of risk behavior and animal injury must be identified to inform interventions to improve the occupational health of rural livestock keepers in Uganda.

## BACKGROUND

As many as 400 million individuals are engaged in rural livestock keeping in Africa.^1^ These keepers rely on livestock for subsistence, to meet social obligations, and to insure against disaster. In Uganda, over 70% of households are engaged in livestock rearing,^2^ many as members of rural pastoralist and agropastoralist communities. In response to a rapid increase in demand for animal-source food, it has been proposed that livestock farming in Africa transition toward intensified management systems to more efficiently use land and resources,^3^ calling into question the role that pastoralism will play in the future of agriculture in Africa.

Livestock keepers are routinely exposed to a variety of hazards. While the hazards of animal husbandry in industrialized settings have been well-documented, including zoonotic diseases^4,5^ and injuries,^6,7^ the risks for rural livestock keepers in Africa remain largely unstudied.

Little has been documented about the hazards traditional livestock keepers face. To better understand the risks and benefits of traditional livestock-keeping systems and describe the occupational practices and hazards of agropastoralist livestock keepers, we conducted an occupational health questionnaire in rural livestock-keeping communities in Moyo, Uganda, from July through September of 2016. The Moyo district has a population of approximately 140,000, 50.4% of whom are female and 55.5% under 17 years of age. Over three-quarters (76.3%) of the population is engaged in livestock farming and two-thirds (68.4%) of the population over 18 years old is literate.^8^

## METHODS

### Study Design and Setting

This study was nested within the larger Syndemic Relationships Among Human Diets and Livestock Associated Zoonotic Diseases study implemented by Veterinarians Without Borders, a cross-sectional study that evaluated the risk factors for and frequency of zoonotic disease transmission in rural livestock keeping communities in Iganga, Arua, Adjumani, and Moyo districts in Uganda. The parent study selected the Iganga district as it was thought to be representative of the Busoga region – a region of interest to local collaborators – while the Arua, Adjumani, and Moyo districts were thought to collectively represent the West Nile Region, a region with high endemicity of the diseases of interest to this study. The parent study collected household survey data relevant to livestock keeping and nutrition as well as human morphometric data and human and cattle tuberculosis skin test data. This survey was administered while parent study data collection was in Moyo District in northwestern Uganda.

### Recruitment of Households

In each district, subcounties, parishes, and villages were identified for sampling by discussion with local government officials, typically district veterinary officers (DVOs), district health officers (DHOs), and/or animal health officers (AHOs). The number of selected subcounties, parishes, and villages varied with the size of the corresponding district and study resources at the time of sampling.

Sampling in Moyo district was conducted in 2 phases, with 2 subcounties sampled in the first phase and 3 in the second. Larger subcounties containing more livestock were preferentially selected in both phases. In the first phase, households within selected subcounties were mobilized to bring their animals to a central location, without specific selection of parishes or villages. A total of 5 parishes and 23 villages were sampled in this phase. In the second phase, 5 parishes and 10 villages were selected, prior to mobilization of households within these villages. Within the selected villages, households were selected and mobilized by the same government official or a colleague, with study administration beginning within a few days of recruitment. All livestock-owning households within a selected village were eligible for participation.

### Data Collection

An animal worker health questionnaire was developed by the authors as a component of the Global Assessment of Zoonotic Exposure Risks (GAZER) study, a multicomponent survey for populations with close contacts with animals. Many of the individual survey items were drawn from previously validated surveys including the National Health and Nutrition Examination Survey^9^ and Patient Health Questionnaire-9.^10^ This component was reviewed by experts prior to piloting (**Appendix A**), and these analyses include several variables from the parent study (**Appendix B**). The tool was piloted in 6 households in Metu subcounty in July 2016. Modifications were made to improve understanding and reduce sensitivity of questions. Questionnaires were prepared in English and administered in Ma'di.

Surveys were completed by in-person interviews with heads of household; all data was self-reported. Administration of the interviews was performed by trained bilingual local members of the study team who simultaneously translated the survey into the local language. We performed all study procedures at participant homesteads after administration of the parent study's survey.

While the questionnaire was primarily comprised of questions that provided only coded check/tick box answers, several questions allowed text entry if the box for “other” was selected. For questions pertaining to delegation of livestock-associated tasks, respondents completed a table identifying tasks commonly performed, the household member usually performing that task (identified by gender and age), and the frequency that the task is performed within the household. With regards to injury variables, respondents were asked about their personal history of needlestick injury and animal-related injuries among household members and in the respective village. Animal injury was defined as any injury caused directly by an animal, such as a bite, kick, or gore, that occurred in the household within the past year.

**Figure:**
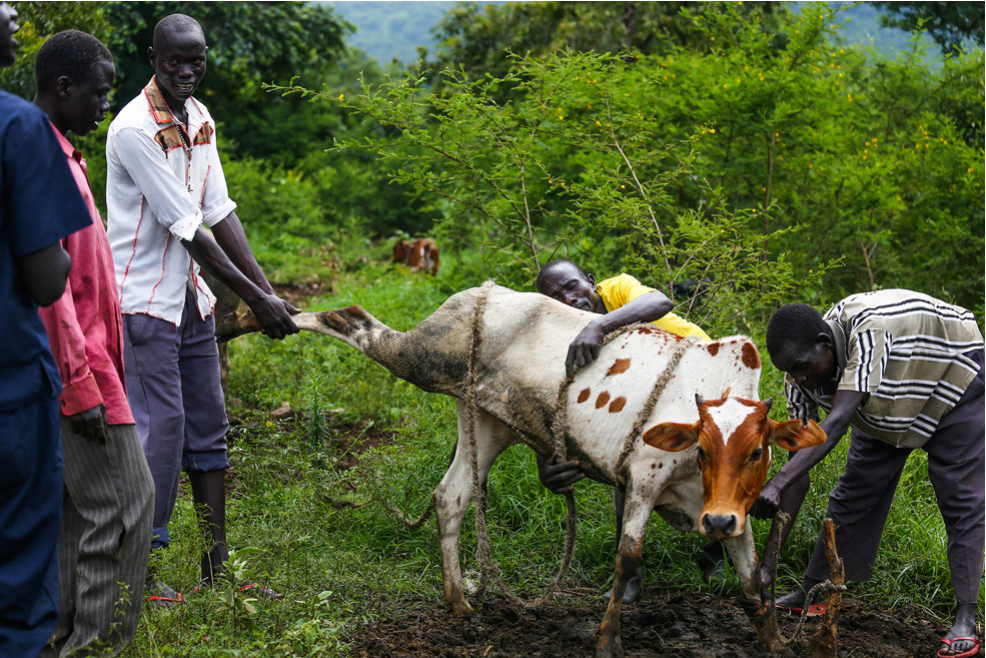
Livestock keepers preparing to cast a cow in Dufile Subcounty, Moyo District. © 2016. J Meisner

### Analyses

We entered completed surveys into Excel (Microsoft Cooperation, Redmond, Washington, USA) and used R v3.2.2 for all further analyses. Univariate analyses were used to generate descriptive statistics for household demographics, herd management practices, delegation of livestock-associated tasks, veterinary care practices, zoonotic disease awareness, personal protective equipment (PPE) use, exposure to animal excreta, and history of needlestick or animal injury. Correlation coefficients were then generated to describe the association between handwashing practices and animal contact as an exploratory analysis.

Logistic regression was used to estimate odds ratios and Wald-type 95% confidence intervals (CI) for risk factors for animal injury, defined as any injury to a household member in the past year that was caused directly by an animal, and adjusted for confounders. Risk factors studied were total number of male animals; number of male cattle, sheep/goats, and pigs; proportion of herd comprised of males; herd size; and castration practices. Potential confounders evaluated – categorized as in **Table 1** – were animal breeds kept (local, local-exotic cross, exotic, or both), household size (number of individuals residing in the household), management system (communal grazing, tethering, combination, or other), addition of new stock within the past year, distance between residence and kraal as perceived by respondent (close, far or very far), respondent education (none, primary, secondary, tertiary, or diploma), respondent occupation (free text), co-housing with animals at night (yes or no), and frequency of animal-associated tasks (mean frequency score over the 9 tasks studied). Confounders were identified via an *a priori* approach. Variables identified *a priori* as independent causes of the outcome of interest – animal injury – were considered as possible confounders, and a directed acyclic graph (DAG) was constructed using DAGitty.net to determine the minimal sufficient adjustment set and avoid over-adjustment^11^; this DAG can be provided on request. Confounders identified in this way were then examined for association with the exposure of interest in the dataset on the basis of correlation coefficients for continuous variables, bivariate frequency tables for nominal categorical variables, and logistic regression coefficients for binary variables.

**TABLE 1.** Demographic and Herd Management Variables From 49 Respondents Corresponding to 49 Households

Variable (missing)	All (N=49) n (%)	Injury Households^a^ (N=22) n (%)	Non-injury Households^a^ (N=27) n (%)
*Individuals*			
Occupation of respondent (2)			
Peasant farmer	46 (97.9)	21 (100)	25 (96.2)
Schoolteacher	1 (2.2)	0 (0)	1 (3.8)
Education level of respondent (2)			
None	4 (8.5)	1 (4.8)	3 (11.5)
Primary	36 (76.6)	16 (76.2)	20 (76.9)
Secondary	4 (8.5)	3 (14.3)	1 (3.8)
Tertiary	2 (4.3)	1 (4.8)	1 (3.8)
Diploma	1 (2.1)	0 (0.0)	1 (3.8)
Hours per day spent caring for livestock (2)	6.5 (2.1)*	6.7 (1.4)*	6.3 (2.6)*
*Households*			
Household size (5)	9.7 (4.7)*	9.3 (4.0)*	10.0 (4.5)*
Herd size (0)	72 (65)*	70.5 (65.6)*	73.3 (65.6)*
Male animals (0)	12.9 (11)*	12.1 (11.9)*	13.6 (11.0)*
Percent of herd comprised of male animals (2)	23 (19)*	22.3 (18.7)*	23.4 (18.7)*
Cattle (3)	41 (46)*	47.1 (57.9)*	36.7 (34.0)*
Male cattle (13)	7.7 (9.9)*	9.4 (14.5)*	6.6 (5.4)*
Sheep and goats (2)	20.3 (32)*	17.1 (18.4)*	23.0 (39.6)*
Male sheep and goats (9)	4.9 (7.4)*	4.5 (5.3)*	5.1 (8.8)*
Pigs (2)	1.8 (2.4)*	1.9 (3.0)*	1.6 (1.7)*
Male pigs (2)	0.9 (1.4)*	1.0 (1.8)*	0.8 (1.1)*
Poultry (2)	12.6 (13)*	10.0 (8.3)*	14.8 (15.6)*
Male poultry (12)	3.3 (3.8)*	2.5 (2.0)*	3.9 (4.6)*
Management (2)			
Communal grazing	40 (85.0)	20 (95.2)	20 (76.9)
Combination	5 (11.0)	1 (4.8)	4 (15.4)
Tether	2 (4.3)	0 (0.0)	2 (7.7)
Breed (2)			
Local	41 (87.0)	20 (95.2)	21 (80.8)
Local–exotic cross	4 (8.5)	1 (4.8)	3 (11.5)
Local and crosses	2 (4.3)	0 (0.0)	2 (7.7)
New stock (2)			
No	33 (70)	13 (61.9)	20 (76.9)
Yes	14 (28)	8 (38.1)	6 (23.1)
Kraal distance (2)^b^			
Far	41 (87)	19 (90.5)	22 (84.6)
Close	6 (13)	2 (9.5)	4 (15.4)
Co-housing with animals at night (3)			
No	45 (98)	21 (100)	24 (96)
Yes	1 (2)	0 (0)	1 (4)
Mean frequency score of livestock-associated tasks^c^	3.4 (0.5)*	3.3 (0.4)*	3.5 (0.5)*

Notes: “Missing” refers to the number of observations for which this variable was not recorded.

* Mean (standard deviation).

a Injury households are those reporting an animal injury to a household member in the past year, as reported by one respondent per household; non-injury households are those reporting no such history.

b Qualitative difference, as perceived by household respondent.

c 1=every day, 2=every week, 3=every month, 4=less than once per month, 5=never.

### Ethical Approvals

The parent study was approved by the Mildmay Uganda Research and Ethics Committee (REC REF 0406-2015) and registered with the Uganda National Council for Science and Technology (approval #1830). Written informed consent was obtained from all participants of the parent study. As completed surveys did not contain any participant identifiers, the Human Subjects Division of the University of Washington determined this activity to constitute “non-engagement” with human subjects.

## RESULTS

### Household Demographics and Herd Management Practices

Questionnaires were administered to 49 households, with a mean size of approximately 10 individuals and a mean herd size of 72 head. Almost all respondents (n=46, 97.9%) were peasant farmers, and most had primary school education (n=36, 76.6%). Local cattle breeds were the most commonly kept (n=41, 87%) and communal grazing was the most commonly-used management system (n=40, 85%). Most (n=41, 87%) of households live “far” from the used kraal, and almost all households (n=45, 98%) did not keep livestock in the home at night. Most respondents (n=33, 70%) have not purchased new stock in the past year. All respondents owned at least 1 male animal, and 45 (97.8%) of respondents owned at least 1 intact (non-castrated) male animal (data not shown). The mean number of male animals kept was 13, or 23% of the herd. The predominant male species kept was cattle, followed by sheep and goats, then poultry, and lastly pigs. On average, survey respondents spend 6.5 hours per day caring for their livestock (**Table 1**).

### Livestock-Associated Tasks

**FIGURE 1.**
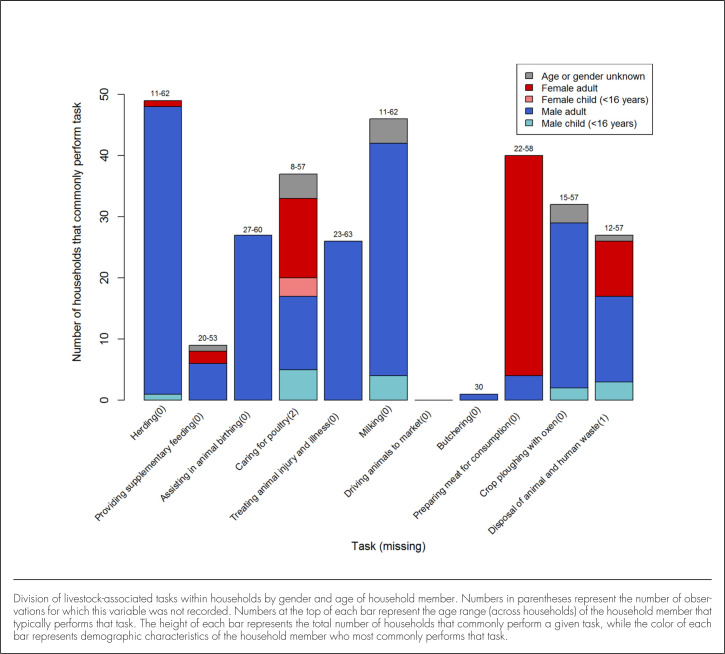
Division of Livestock-Associated Tasks Within Households (N=49)

Most livestock-associated tasks were performed by adult men, including herding (n=47, 95.9% of households that commonly perform this task), assisting in animal birthing (n=27, 100%), treating injured or ill animals (n=26, 100%), milking animals (n=38, 82.6%), and ploughing crops with oxen (n=27, 93%). Women and girls more commonly provided care for poultry, while adult women also prepared meat for home consumption, disposed of animal and human waste, and provided supplementary feeding to livestock. Male children were reported to herd livestock to grazing sites, provide care for poultry, milk cattle, perform crop ploughing using animal traction, and dispose of human and animal waste (**Figure 1**).

Division of livestock-associated tasks within households by gender and age of household member. Numbers in parentheses represent the number of observations for which this variable was not recorded. Numbers at the top of each bar represent the age range (across households) of the household member that typically performs that task. The height of each bar represents the total number of households that commonly perform a given task, while the color of each bar represents demographic characteristics of the household member who most commonly performs that task.

Most households reported they care for poultry and milk cattle daily, move herd livestock to grazing areas weekly, and prepare meat for home consumption less than once per month. Almost a quarter (n=22, 45%) of households reported they do not assist in animal birthing, while almost two-fifths (n=19, 39%) perform this task less than once per month. More than a third (n=17, 36%) of households reported they do not plough crops with animal traction, while less than a third (n=15, 32%) perform this task weekly. Almost half (n=21, 45%) of households do not dispose of waste, while over a third (n=17, 36%) perform this task daily. Most households did not provide supplementary feeding (n=40, 82%), treat animal injury or illness (n=23, 47%), drive animals to market (n=49, 100%), or butcher animals (n=48, 98%) (**Table 2**).

**TABLE 2.** Frequency at Which Households Report Performing Livestock-Associated Tasks

	Number of Households (%)				
Task (missing)	Not Performed	Daily	Weekly	Monthly	Less Than Once per Month
Herding (0)	0 (0.0)	17 (35.0)	25 (51.0)	6 (12.0)	1 (2.0)
Supplementary feeding (0)	40 (82.0)	3 (6.1)	3 (6.1)	2 (4.1)	1 (2.0)
Assist in animal birthing (0)	22 (45.0)	1 (2.0)	0 (0.0)	7 (14.0)	19 (39.0)
Caring for poultry (2)	10 (23.0)	34 (77.0)	0 (0.0)	0 (0.0)	0 (0.0)
Treating animal injury and illness (0)	23 (47.0)	1 (2.0)	5 (10.0)	8 (16.0)	12 (24.0)
Milking (0)	4 (8.3)	26 (54.0)	14 (29.0)	3 (6.3)	1 (2.1)
Driving animals to market (0)	49 (100.0)	0 (0.0)	0 (0.0)	0 (0.0)	0 (0.0)
Butchering (0)	48 (98.0)	0 (0.0)	0 (0.0)	0 (0.0)	1 (2.0)
Preparation of meat for home consumption (0)	9 (19.0)	4 (8.5)	7 (15.0)	11 (23.0)	16 (34.0)
Crop ploughing with oxen (0)	17 (36.0)	8 (17.0)	15 (32.0)	6 (13.0)	1 (2.2)
Disposal of human and animal waste (1)	21 (45.0)	17 (36.0)	5 (11.0)	0 (0.0)	4 (8.5)

Notes: “Missing” refers to the number of observations for which this variable was not recorded. Printed surveys defined these tasks as detailed in **Appendix A**. Columns denote the number and percent of households that perform a given task at a given frequency.

### Hygiene Practices and Exposure to Potentially Infectious Material

Most respondents (n=28, 57%) commonly use PPE – typically gumboots. With regard to handwashing, 41 (85.4%) respondents reported they routinely wash their hands; of this group, 31 (75.6%) using soap and water, 8 (19.5%) using water only, and 2 (4.9%) using another substance, such as ash (**Table 3**). All respondents reported they “often” wash their hands before eating (data not shown). Most respondents report handwashing as “often” after milking (n=41, 89%), before handling meat (n=29, 63%), after processing meat (n=21, 62%), after assisting with animal birth (n=18, 58%), and after handling blood, urine, or manure (n=24, 57%). Respondents less commonly washed hands before drinking (sometimes/rarely: n=22, 45%; never: n=12, 25%), after butchering animals (sometimes/rarely: n=3, 33%; never: n=2, 22%), and after touching animals (sometimes/rarely: n=32, 70%; never: n=3, 6.5%). Note, all frequency measures such as “routinely” and “often” were defined by the respondent.

**TABLE 3.** Handwashing and PPE Practices As Reported By One Respondent Per Household

Behavior (missing)	n (%)
PPE use (0)	
Commonly used	28 (57)
Gumboots	14 (50.0)
Gumboots and raincoat	5 (21.4)
Gumboots and overalls	4 (14.3)
Raincoat	3 (10.7)
Gumboots, overalls, and raincoat	1 (3.6)
Not commonly used	21 (43)
Handwashing (1)	
Typically done	41 (85.4)
Soap and water	31 (75.6)
Water only	8 (19.5)
Other (typically with ash)	2 (4.9)
Typically not done	7 (14.6)

Note: “Missing” refers to the number of observations for which this variable was not recorded.

Respondents reported they “often” had contact with faeces (n=38, 78%), and “sometimes/rarely” had contact with blood (n=25, 76%), urine (n=30, 61%), animal flesh (n=39, 81%), and/or animal fluids (n=31, 63%) (data not shown). Both general handwashing practices (commonly performed yes/no) and frequency of handwashing after animal contact were positively and significantly associated with frequency of contact of blood (general handwashing: correlation coefficient [r]=0.32; 95% CI, 0.03 to 0.55 and handwashing after animal contact: r=0.36; 95% CI, 0.09 to 0.57) and animal fluids (general handwashing: r=0.33; 95% CI, 0.05 to 0.56 and handwashing after animal contact: r=0.36; 95% CI, 0.09 to 0.59), although this association was not strong (**Table 4**).

**TABLE 4.** Correlation Between Handwashing Practices and Frequency of Contact With Animal Excreta, Expressed As Pearson's Product Moment Correlation Coefficient

	Blood (r, 95% CI)	Urine (r, 95% CI)	Faeces (r, 95% CI)	Flesh (r, 95% CI)	Fluids (r, 95% CI)
General handwashing	0.32 (0.03, 0.55)	0.24 (-0.05, 0.50)	-0.07 (-0.34, 0.22)	-0.10 (-0.38, 0.19)	0.33 (0.05, 0.56)
After animal contact	0.36 (0.09, 0.58)	0.17 (-0.11, 0.43)	0.21 (-0.07, 0.47)	-0.21 (-0.40, 0.07)	0.36 (0.09, 0.59)

Notes: General handwashing defined by yes (0) vs. no (1) answer to the question “Do you routinely wash hands after touching your animals”. Handwashing after animal contact defined by categorical answer to the question “How often do you wash your hands after touching an animal”: often (1), sometimes (2), rarely (3), never (4), not applicable (5). Abbreviations: CI, confidence interval; r, correlation coefficient.

Most respondents (n=28, 57.2%) believed diseases could be transmitted from animals to humans, however, only half of these 28 believed transmission could go in the reverse – from humans to animals. Most did not believe that sick animals can look healthy (n=25, 53.2%), and almost all believed they can tell when their animals are sick (n=48, 98%). In 31 (63%) households, respondents reported that a veterinary professional provides care for their livestock, while in the remaining households, a household member provides veterinary care (n=15, 31%) or both household members and veterinary professionals provide veterinary care (n=3, 6%). It is important to note that these data did not distinguish between preventative care versus care for sick animals, nor frequency at which such care was provided. In households where a household member provides veterinary care, all reported that an adult male household member (mean age 44.3 years old, range 23 to 76 years) performs this task (data not shown).

### Needlestick and Animal Injury

Six (12%) respondents – or 40% of the 15 respondents residing in a household in which a household member provides veterinary care – reported any history of a needlestick injury to themselves (**Table 5**), without time bounds. Twenty-two (45%) households report injury to household members caused by animals within the past year, with most injuries being identified as gores (54.5%) (**Table 5**). Four households (8.2%) reported more than 1 injury within the past year: 2 muscular strains, 1 gore, and 1 scratch; and 1 household reported 4 injuries in the past year. In all households with a history of injury, the injured household member was male, with mean age of 38 years old and age range of 10 to 60 years. Most injuries were caused by cattle (86.0%) and female animals (77.8%) (**Table 5**). A total of 7 respondents reported any household history of serious injury, with 6 of these being open wounds, all caused by cattle, and most caused by female animals (n=4, 80%) (**Table 5**). Sixteen households reported knowledge of serious animal injury in the village, with 12 of these being open wounds, and most caused by cattle (n=11, 78.6%); however, only half of those injuries were caused by female animals (**Table 5**).

**TABLE 5.** History of Needlestick Injury or Animal Injury, as Reported by One Respondent Per Household

Injury (missing)	n (%)
Needlestick (0)	
Never	32 (65.3)
Do not give injectable medications to animals	10 (20.4)
Ever	6 (12.2)
Don't know	1 (2.0)
Animal: any, household, this year (0)	
No	27 (55)
Yes	22 (45)
Injury type (0)	
Gore	12 (54.5)
Muscular strain	6 (27.5)
Bite	2 (9.1)
Scratch	1 (4.5)
Crush	1 (4.5)
Animal species (0)	
Bovine	19 (86.0)
Swine	2 (9.1)
Canine	1 (4.5)
Animal sex (4)	
Female animal	14 (77.8)
Male animal	4 (22.2)
Animal: serious, household, ever	
Injury (4)	
None	38 (84.4)
Open wound/no death	6 (13.3)
Fracture/no death	1 (2.2)
Death	0 (0.0)
Animal species (2)	
Bovine	5 (100)
Animal sex (1)	
Female	4 (80)
Male	2 (20)
Animal: serious, village, ever	
Injury (7)	
None	28 (61.9)
Open wound/no death	12 (28.6)
Fracture/no death	3 (7.1)
Other	1 (2.4)
Death	0 (0.0)
Animal species (2)	
Bovine	11 (78.6)
Canine	3 (21.4)
Animal sex (2)	
Female	7 (50)
Male	7 (50)

Note: “Missing” refers to the number of observations for which this variable was not recorded.

### Regression

After adjustment for confounders, the odds of animal injury in the household in the past year was higher in households that kept a greater number of male pigs (OR 1.05; 95% CI, 0.94 to 1.17), in households that kept a herd with a greater proportion of males (OR 1.33; 95% CI, 0.25 to 7.02), and in households that ever versus never castrated male animals (OR 1.15; 95% CI, 0.72 to 1.83), however, none of these associations were statistically significant (**Table 6**). These data did not provide any other evidence of association between the exposures studied and animal injury.

**TABLE 6.** Association Between Herd Management Variables and Any Animal-Associated Injury in the Household in the Past Year

	Adjusted	
	OR	95% CI
Number of males kept	1.00^a^	0.99, 1.02^a^
Number of male cattle	0.99^b^	0.96, 1.03^b^
Number of male sheep/goats	0.99^c^	0.96, 1.02^c^
Number of male pigs	1.05^d^	0.94, 1.17^d^
Proportion male	1.33^e^	0.25, 7.02^e^
Herd size	1.00^f^	1.00, 1.00^f^
Castration	1.15^g^	0.72, 1.83^g^

a Adjusted for herd size, number of household residents, breed

b Adjusted for herd size, breed

c Adjusted for herd size, number of household residents, co-housing with animals at night

d Adjusted for new stock

e Adjusted for number of males, herd size, co-housing with animals at night

f Adjusted for number of male animals, number of household residents

g Adjusted for proportion males, breed, management system, occupation

Abbreviations: CI, confidence interval; OR, odds ratio.

## DISCUSSION

Our study found animal injury and needlestick injury to be common, task delegation to be distinct between men and women and adults and adolescents, contact with potentially infectious material to be common, and both handwashing with soap and glove use to be uncommon. Furthermore, while rural livestock keepers in Uganda appear to be generally familiar with zoonoses, most do not recognize the zoonotic risk of subclinical infections.

Most of the previous studies on rural livestock-keeping communities in Africa have focused their research on zoonotic diseases,^12^ with minimal attention paid to work practice-related risks. An exception to this is a 2-part review of the occupational risks of livestock and crop farmers in The Gambia by Kuye et al.^13,14^ In their report, 80% of farmers reported a work-related injury in the past year, which was far higher than our results. While our study did not ask specifically about whether animal injuries were incurred in the context of work, we considered all animal keepers as workers, and it is reasonable to assume animal injuries did not occur outside of the context of animal keeping. While our survey asked specifically about animal-origin and needle-stick injury – rather than all work-related injuries – our animal-injury question pertained to the entire household and our needlestick-injury question did not define time bounds, suggesting questionnaire differences should result in higher reporting in our study. It is not possible to conclude if this discordance can be explained by questionnaire structure, by biases in 1 or both studies, or by differences between Gambian and Ugandan farmers.^12–14^

To our knowledge, there have been no previous efforts to describe the frequency with which livestock-associated tasks are performed in this setting, though limited prior efforts have been made to describe the delegation of livestock-associated tasks within households in rural African communities. A review of literature from Zimbabwe found that men are usually responsible for outside work and women are responsible for inside work and the feeding of animals; domestic fowl are mainly owned by women and boys are responsible for milking and herding animals.^18^ This is largely consistent with our finding that the majority of livestock-associated tasks are completed by men, with women and girls caring for poultry, feeding livestock, and performing household-related tasks, and with boys herding livestock and milking cattle. However, our study also found that boys may also perform crop ploughing, provide care for poultry, and dispose of human and animal waste, suggesting there is not a clear delineation between tasks commonly performed by women and girls and those performed by boys. Notably, our study found caring for poultry, milking cattle, and herding livestock to be the most commonly performed tasks, suggesting that women and children frequently contact livestock. While these data do not allow conclusions to be drawn regarding specific hazards arising from this contact, they do suggest that future efforts to enumerate exposure to animal-related hazards in this setting should include women and adolescents.

Despite the fact that all respondents keep male animals, and almost all keep intact/non-castrated male animals, most injuries were caused by female animals and cattle. This may be due either to greater contact with female cattle than other animals, or to behavioral differences between male and female animals. No evidence was found to suggest that keeping of male animals, castration practices, and herd size are risk factors for animal injury, in contrast with findings from the United States.^6^ This may be the result of temperamental differences in Ugandan vs. U.S. male livestock – due to breed or husbandry differences – or biases in study design. It is not unreasonable to hypothesize that in a setting where male animals are kept with the female and juvenile herd and handled frequently, they are more socialized and less dangerous, although, to our knowledge, no studies exist on male animal behavior in a rural African livestock keeping setting. While this study found that most victims of injury were men, livestock development interventions are increasingly targeting women to alleviate gender disparities^15^ and promote agricultural development; as livestock keeping transitions towards women, women may be at greater risk of animal injury.

This study showed that rural livestock keepers in Uganda are commonly exposed to animal faeces, and that while most respondents wash their hands regularly, nearly one-quarter of respondents do not use soap and most do not wash their hands after touching animals, putting them at risk for zoonoses transmitted through the animal faeces. While over half report PPE use, none of the PPE types listed provide respiratory and mucous membrane protection, and gloves are not worn. Compounding this lack of rigorous PPE use and hygiene standards is the finding that over half of our respondents did not believe that sick animals may look healthy. While most respondents were aware of zoonoses, and almost two-thirds use professional veterinary services to provide care for their livestock, this knowledge and practice will not prevent transmission of zoonoses, such as brucellosis, from animals that have no clinical signs.

### Limitations

There are several limitations to this study. The sample size was small, with only 49 households surveyed. All analyses were complete case, meaning that observations or variables with missing data were dropped from analyses. This could introduce bias if missingness is not completely at random, that is if missingness is the result of observed or unobserved variables. While less than 5% of observations were missing for most variables, higher multivariate missingness is a concern in the regression models. Furthermore, even if missingness is completely at random, the loss of observations with missing data compromises the already limited precision of this study. Additionally, households were selected by convenience sampling. This may have introduced selection bias if the households selected to participate differed in their practices or exposures than those not selected. It should be noted that household recruitment was not specific to this questionnaire, but to the parent study, and, as a result, potential bias arising from this is difficult to quantify.

Information bias may arise due to the self-reported nature of these data and the need to administer this questionnaire in the Ma'di language. While the questionnaire was reviewed by Ugandan and U.S. experts in occupational health and veterinary medicine, validation of translation was not performed. While an author was present for questionnaire administration and able to answer translator questions as they arose, formal cognitive interviewing of respondents or translators was not performed. Information bias may also result from the household-level nature of questionnaire administration, as only 1 member of each household – typically the head of household – completed the questionnaire. This imparts a multilevel quality to these data, as some questions pertained to household practices and exposures while others pertained to the individual's exposures and practices. Where the individual is answering for the household, potential biases may arise if the individual's understanding of other household members' practices or exposures is inaccurate. Typically, other household members were present while the questionnaire was administered, but we are not certain to what extent these individuals were consulted for answers. It is difficult to predict the possible direction of these resultant biases, thus bias away from the null or to the other side of the null cannot be ruled-out.

Finally, the regression analyses conducted inherit the limitations of any cross-sectional study, which is an uncertain temporal relationship between outcome (animal injury) and the exposures (herd composition and castration practices). As it seems unlikely that a household's history of animal injury causes changes in herd composition or castration practices, we do not think this is an important limitation.

## CONCLUSION

Currently, traditional livestock-keeping systems predominate in Uganda, with 16 times more workers engaged in the subsistence agricultural sector than the commercial sector.^2^ However, agriculture in Africa is trending toward intensified management systems – where more animals are kept per unit area and per worker – and the inclusion of women, and away from male-dominated traditional livestock-keeping systems. With this change will likely come an intensification of the hazards already present and extension of these hazards into previously unexposed demographic groups. In Uganda, the agricultural sector is within the purview of the Occupational Safety and Health Act, 2006 (No 9).^16^ This study demonstrates that despite this appropriate legislative framework, agricultural injury and illness are common. These findings should motivate future research efforts to identify reasons for injury and illness and predict the impact of intensification on the health of rural livestock keepers, so that effective interventions can be developed and targeted to appropriate worker groups. Collaboration with qualitative researchers should be sought to better understand what tasks performed, describe how they are performed, and understand the drivers for task delegation and risky behaviors, such as low PPE use. Livestock keeping brings wealth, means of transport, financial security, and animal-source nutrition to rural communities. However, with these benefits come the necessity of providing animal husbandry and the attendant risks of this work. When considering the future of agriculture in Africa, attention must not be solely focused on food security and economic development at the expense of the health of communities that would otherwise benefit from these gains.
